# Influence of Cold-TRP Receptors on Cold-Influenced Behaviour

**DOI:** 10.3390/ph15010042

**Published:** 2021-12-28

**Authors:** Dibesh Thapa, Brentton Barrett, Fulye Argunhan, Susan D. Brain

**Affiliations:** Section of Vascular Biology and Inflammation, School of Cardiovascular Medicine and Sciences, BHF Centre of Research Excellence, Franklin-Wilkins Building, Waterloo Campus, King’s College London, London SE1 9NH, UK; dibesh.thapa@kcl.ac.uk (D.T.); brentton.barrett@kcl.ac.uk (B.B.); fulye.argunhan@kcl.ac.uk (F.A.)

**Keywords:** TRPA1, TRPM8, cold, thermoregulation, UCP-1, BAT

## Abstract

The transient receptor potential (TRP) channels, TRPA1 and TRPM8, are thermo-receptors that detect cold and cool temperatures and play pivotal roles in mediating the cold-induced vascular response. In this study, we investigated the role of TRPA1 and TRPM8 in the thermoregulatory behavioural responses to environmental cold exposure by measuring core body temperature and locomotor activity using a telemetry device that was surgically implanted in mice. The core body temperature of mice that were cooled at 4 °C over 3 h was increased and this was accompanied by an increase in UCP-1 and TRPM8 level as detected by Western blot. We then established an effective route, by which the TRP antagonists could be administered orally with palatable food. This avoids the physical restraint of mice, which is crucial as that could influence the behavioural results. Using selective pharmacological antagonists A967079 and AMTB for TRPA1 and TRPM8 receptors, respectively, we show that TRPM8, but not TRPA1, plays a direct role in thermoregulation response to whole body cold exposure in the mouse. Additionally, we provide evidence of increased TRPM8 levels after cold exposure which could be a protective response to increase core body temperature to counter cold.

## 1. Introduction

Mammals possess several mechanisms to adapt to temperature changes to preserve physiological homeostasis and conserve core body temperature [1]. One example of such a mechanism that is involved in maintaining core body temperature when exposed to the cold is the cutaneous vasoconstriction response that is often clearly observed in the extremities of the body such as the fingers and toes [1,2]. The body induces further protective mechanisms against the cold to maintain core body temperature; shivering and non-shivering thermogenesis. When cold stimuli are detected by the body, the sympathetic nervous system [3] activates non-shivering thermogenesis in the brown adipose tissue (BAT) to produce heat by upregulation of uncoupling protein 1 (UCP-1) which acts to uncouple oxidative phosphorylation and inhibit ATP production, leading to cellular heat [4,5]. Within the past 20 years, the understanding of cold sensation via cell sensors and receptors has advanced, but it has only been within the last decade that studies have been conducted to link the cold sensors with thermogenesis. The study of transient receptor potential (TRP) channels has been central to this and we investigate this further here.

TRP channels are non-selective cation channels which act as polymodal sensors and are involved in a range of physiological functions [6]. TRPA1 is a cold sensor that was discovered when a steady decrease in temperature from 25 °C stimulated an increase in cytosolic calcium levels at 17 °C, with the highest magnitude at 11 °C in TRPA1 transfected Chinese hamster ovary (CHO) cells [7]. The pharmacological inhibition with TRPA1 antagonists and the absence of extracellular calcium all abolished the sensitivity to cold [7]. Since then, there has been debate on the role of TRPA1, with it being suggested that TRPA1 does not act as a cold sensor for autonomic thermoregulation in rodents from study of exposure to the cold [8] but is highly active in skin after local environmental cooling, in mice at least [9].

TRPM8 was discovered in 2001 [10] and confirmed as a cold-sensitive TRP channel before TRPA1. However, there have been multiple studies debating the exact threshold for TRPM8. There are two distinct studies that show that temperatures below 30 °C and 23 °C induced increased currents which were mediated by TRPM8, yet a later study showed a threshold for 22 °C [11,12,13]. Thus, TRPM8 responds to milder temperatures which are deemed innocuous in comparison to the noxious temperature range of TRPA1. It was confirmed in in vitro studies using trigeminal ganglia, in which the calcium influx that was induced by 18 °C cold was absent in tissues from the TRPM8 KO mice [14]. Studies have observed cold sensation in WT and transgenic TRPM8 KO mice in investigation of behavioural responses to a range of temperatures (0–20 °C) [14,15,16]. Of note, the direct activation of TRPM8 using a topical application of the TRPM8 agonist menthol increases heat-seeking behaviours [17,18]. Overall, it is generally agreed that TRPM8 plays an important role in determining the temperature preference in vivo, potentially acting as a thermoreceptor [17,18]. Additionally, TRPM8 has been shown to allow mice to respond to different temperatures, helping with adaptation [19]. More recently, it was found that there is an evolutionary adaption component of TRPM8 in response to cold climates [20]. TRPM8 from penguins had a remarkably high tolerance for cold in comparison to the African elephant [20].

The role of TRPM8 in thermogenesis is of interest as increased thermogenesis is linked to weight loss. An early finding showed that menthol stimulated-BAT cells and dietary consumption of menthol led to the upregulation of the key thermogenic protein, UCP-1, in WT but not TRPM8 KO mice [21]. However, few in vivo studies to date have investigated the direct link between cold-sensitive TRP channels with cold-induced temperature regulation and thermogenesis in vivo. Early evidence revealed that a TRPM8 blockade leads to a decrease in body temperature but results since have been more mixed [22]. This is possibly linked to TRPM8 KO, when compared to WT mice, possessing lower UCP-1 expression levels [23] although the genetic deletion of TRPM8 does not influence the basal core body temperature. More recently though, a single oral administration of five different selective TRPM8 antagonists has been shown to significantly reduce the core body temperature in mice [24]. Additionally, a study that was aimed at understanding the mechanisms that are involved in hypothermia, for therapeutic reasons, showed that a TRPM8 antagonist had no effect on 10 °C cooling of mice but inhibited the maintenance of body temperature during short mild cooling (18 °C) in a study involving temporary anaesthesia [25]. A later study showed that a TRPM8 antagonist in aged mice caused enhanced hypothermia [26]. Additionally, TRPM8 KO mice have shown an impaired ability to maintain their core body temperature in response to the cold, which was observed alongside increased peripheral heat loss through the mouse tail [27], a thermoregulatory organ in rodents [28]. Overall, it is established that TRPM8 is clearly involved in cold sensing and has the ability to adapt to the ambient temperature within the environment. Perhaps surprisingly, TRPM8 has also been linked to sensing warm temperatures in mice with authors suggesting that a dual sensory model is required in skin [29].

It is well established that cold exposure upregulates the expression of UCP-1 in BAT to induce thermogenesis and to generate heat to counter the cold stimuli. Here, we investigated the link between these two signalling pathways as cold exposure occurs and compare the effect of TRPA1 and TRPM8 antagonists. TRPM8 has been found to induce thermogenesis via menthol stimulation through UCP-1 [21]. However, the relationship between direct cold exposure with TRPM8 and UCP-1 upregulation in BAT and the influence on thermogenesis in response to environmental cold exposure is unclear.

The specific objectives of this study were to; (i) investigate the core body temperature changes in response to environmental cold exposure in comparison to room temperature; (ii) examine the protein expression of UCP-1, a key modulator of thermogenesis, and TRPM8 in mice that were exposed to environmental cold; and (iii) use pharmacological antagonists of TRPA1 and TRPM8 to decipher the activity of these TRP channels in thermogenesis.

## 2. Results

### 2.1. Cold Induces Increased Core Body Temperature

**Figure 1:**
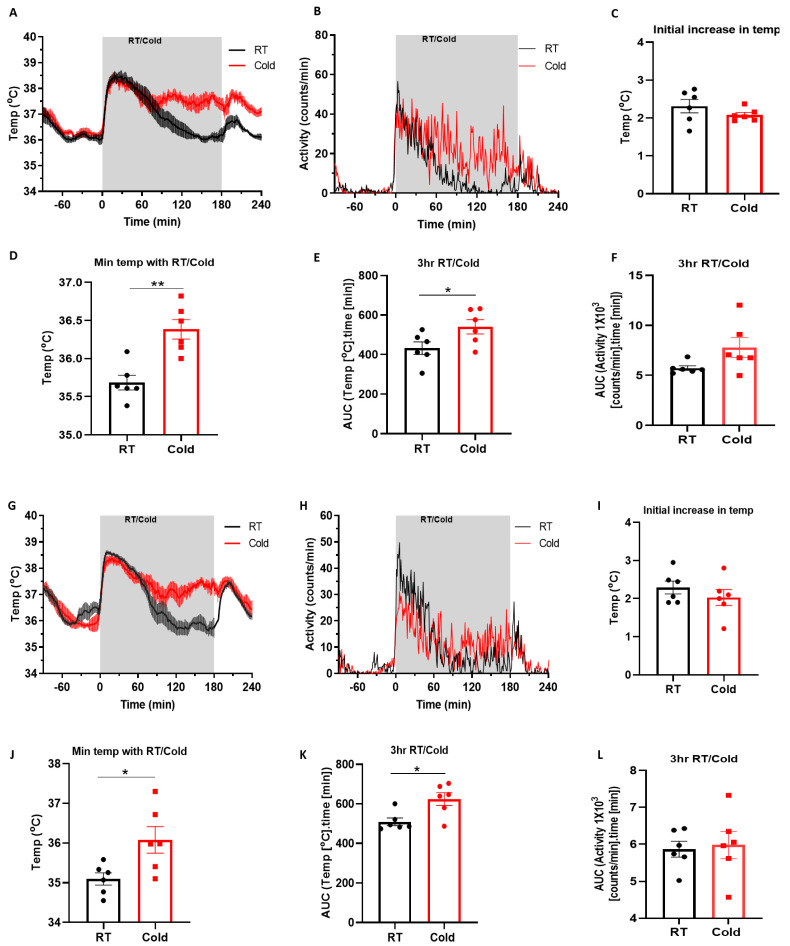
The effect of environmental cold in two different mice strains (**A**–**F**) Graphs for the CD-1 mice represent (**A**) the mean core body temperature and (**B**) the mean activity changes in the CD1 mice during 3 h of cold (4 °C) exposure in a climatic chamber or room temperature treatment (RT) (*n* = 6). (**C**) the initial temperature increase at the start of cold exposure in climatic chamber or RT treatment; (**D**) the minimum temperature that was achieved during 3 h of RT/cold exposure; (**E**) the area under the curve (AUC) analysis of temperature during 3 h of RT/Cold treatment; (**F**) the area under the curve (AUC) analysis of the activity during 3 h of RT/Cold treatment. The results are for *n* = 6 mice. (**G**–**L**) Graphs for C57BL/6 mice represents (**G**) the mean core body temperature and (**H**) the mean activity changes in mice during 3 h cold (4 °C) exposure in a climatic chamber or RT treatment (*n* = 6). (**I**) the initial temperature increase at the start of cold exposure in the climatic chamber or RT treatment; (**J**) the minimum temperature that was achieved during 3 h of RT/cold exposure; (**K**) The area under the curve (AUC) analysis of temperature during 3 h of RT/cold treatment. (**L**) The area under the curve (AUC) analysis of activity during 3 h of RT/cold treatment. The results are for *n* = 6 mice. All the data are presented as the mean and the error bars indicate standard error of mean (S.E.M). * *p* < 0.05, ** *p* < 0.01; Two-tailed Student’s *t*-test.

The mice for experiments involving the cold climatic chamber underwent surgery for the implantation of telemetry devices to enable an accurate measurement of the core body temperature and locomotor activity. The individual home cages containing the telemetered mice with the receiver pads below allowed continuous measurement. There were two strains of mice that were used that revealed similar data (Figure 1A–F for CD-1 mice and Figure 1G–L for C57BL/6J mice). As the mice settled, a stable baseline temperature, (approximately 36 °C) was observed. The movement into the climatic chamber for the room temperature (RT) or the cold (4 °C) treatment was associated with a rapid increase in temperature (Figure 1A,C,G,I) and an associated increase in activity (Figure 1B,H) in both the strains of mice. The mice that were exposed to the cold temperature maintained a higher temperature throughout the 3 h cold period (Figure 1E,K) and had a significantly higher minimum temperature (36.3 ± 0.1 °C vs. 35.6 ± 0.1 °C in CD1, *p* < 0.01, Figure 1D) and (36.1 ± 0.3 °C vs. 35.1 ± 0.1 °C, *p* < 0.05 in C57, Figure 1J) compared to the mice that were exposed to the room temperature without being hypothermic. The locomotor activity, measured using the telemetry probe, was analysed for statistical differences. Neither the CD1- nor the C57BL/6-strain mice showed any significant difference in activity in the cold compared to the room temperature treatment (Figure 1F,L).

### 2.2. Cold Upregulates UCP-1 and TRPM8

**Figure 2:**
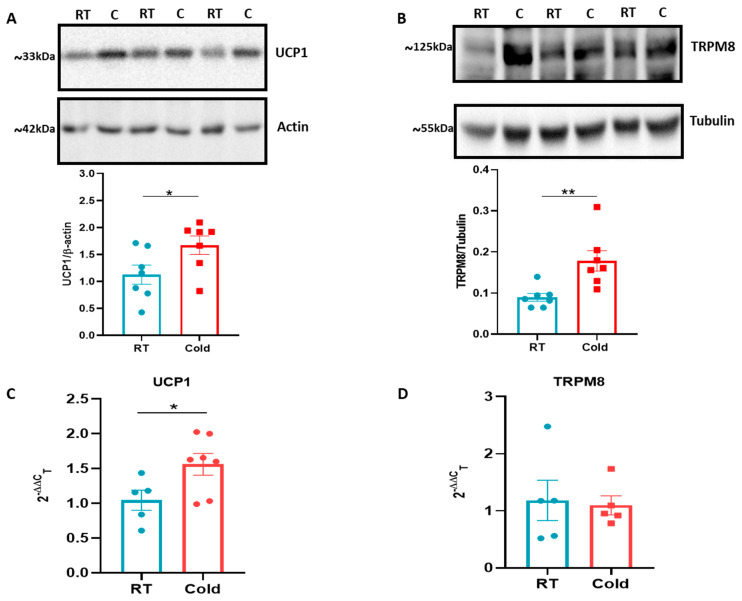
The effect of environmental cold on UCP-1 and TRPM8 in brown adipose tissue (BAT) of C57BL/6 mice; (**A**–**D**) The representative Western blot of UCP-1 (**A**) and TRPM8 (**B**) in BAT with 3-h of cold (4 °C) exposure in a climatic chamber and the respective densitometric analysis normalised to a housekeeping protein. RT-PCR CT analysis shows fold change of UCP-1 (**C**) and TRPM8 (**D**) normalized to three housekeeping genes in BAT. All the data are presented as the mean and error bars indicate S.E.M. (*n* = 5–7), * *p* < 0.05, ** *p* < 0.01. (Two-tailed Student’s *t*-test).

The whole-body cold exposure was associated with an increase in UCP-1 expression in BAT, as expected. We show this via both a Western blot and RT-PCR (Figure 2A,C). However, interestingly our results showed that the cold exposure also caused an increase in BAT TRPM8 protein expression, as determined by Western blot (Figure 2B), suggesting TRPM8 plays an important reactive role in thermogenesis in BAT, although we did not observe this via RT-PCR (Figure 2D). We do not have access to a selective TRPA1 antibody that passes scrutiny tests and were unable to investigate TRPA1 protein levels.

### 2.3. A967079 and AMTB Inhibits TRPA1 and TRPM8 Response

The selective TRPA1 antagonist A967079 and the selective TRPM8 antagonist (AMTB) are traditionally given via intraperitoneal injection in in vivo studies, however, this involves animal handling which creates stress for the animals [30,31]. We selected to establish a system by which the antagonists could be given orally in a stress-reduced manner, to minimize the effect of physical stress on the animals’ core body temperature. Mice rapidly eat the chocolate spread ‘Nutella’, hence, we utilised oral administration of the drug [32] whereby the TRPA1 and TRPM8 antagonists could be given on a Nutella spread and rapidly consumed. We investigated the TRPA1 antagonist in addition to the TRPM8 antagonist for comparative reasons.

**Figure 3:**
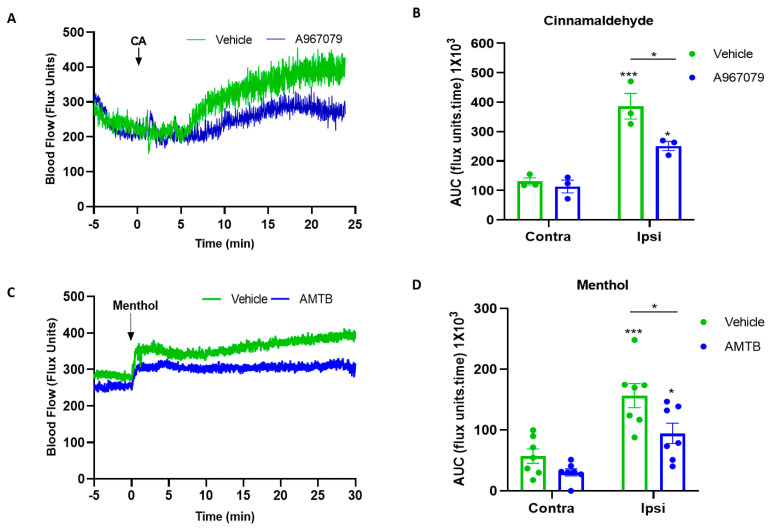
Determining an effective oral dose of the TRP antagonist given in Nutella spread in the CD1 mouse. (**A**) The graph shows the mean blood flow response to the topical application of the TRPA1 agonist cinnamaldehyde (CA, 10%) on the ipsilateral ear in the presence of the TRPA1 antagonist, A967079 (100 mg/kg), given orally 30 min prior to CA application. (**B**) The area under the curve (AUC) analysis of the blood flow increase after vehicle (10% DMSO) or CA treatment in the presence of A967079 (*n* = 3). (**C**) The graph shows the mean blood flow response to the topical application of the TRPM8 agonist menthol (10%) on the ipsilateral ear in the presence of the TRPM8 antagonist, AMTB (10 mg/kg), given orally 30 min prior to menthol application. (**D**) The area under the curve (AUC) analysis of the blood flow increase after vehicle (10% DMSO) or menthol treatment in the presence of AMTB (*n* = 7). (All the data are shown as mean and error bars indicate S.E.M. * *p* < 0.05, *** *p* < 0.001. (Two-way ANOVA, Tukey’s post hoc test).

To determine the effectiveness of this methodology, we needed to ensure that a typical TRPA1 response was inhibited by oral TRPA1 antagonist treatment. We investigated the effect of the TRPA1 agonist cinnamaldehyde (CA) on the mouse ear, which we have previously shown to cause increased blood flow, mediated by vasodilators, primarily the neuropeptide calcitonin-gene related peptide (CGRP) [33]. A967079 (100 mg/kg) was given in Nutella spread 30 min before the start of the blood flow recording, which mice consumed immediately. After 5 min of basal blood flow recording, the topical administration of CA was applied on the ear of the mouse. The vasodilator response to CA was observed from about 5 min after the topical application and continued for the duration of the 30 min measuring period, as expected. This response was diminished in the presence of A967079 (Figure 3A) compared to the vehicle-treated mice which showed a normal response. The vehicle treatment (10% DMSO, 10% tween) had no effect on the blood flow, however, A967079 treatment significantly inhibited the response when using a minimal mouse number (in keeping with the 3Rs). The result is also shown as an area under the curve analysis (Figure 3B) and shows a significant inhibition with this dose of A967079. Next, we tested the effect of the TRPM8 antagonist AMTB (10 mg/kg) when given orally via Nutella spread 30 min before the recording, similar to A967079. To investigate this, we used a vasoactive TRPM8 agonist menthol and its effect on ear blood flow. Similarly to CA, the topical application of menthol induced an increased blood flow, however, this response was blunted by AMTB pretreatment (Figure 3C). The vehicle treatment had no effect on the blood flow, however, AMTB treatment significantly inhibited the response. This is also shown by an area under the curve analysis where AMTB significantly blunts the response of menthol (Figure 3D). The vehicle treatment did not cause any adverse effects.

### 2.4. TRPM8 but Not TRPA1 Influences Core Body Temperature

**Figure 4:**
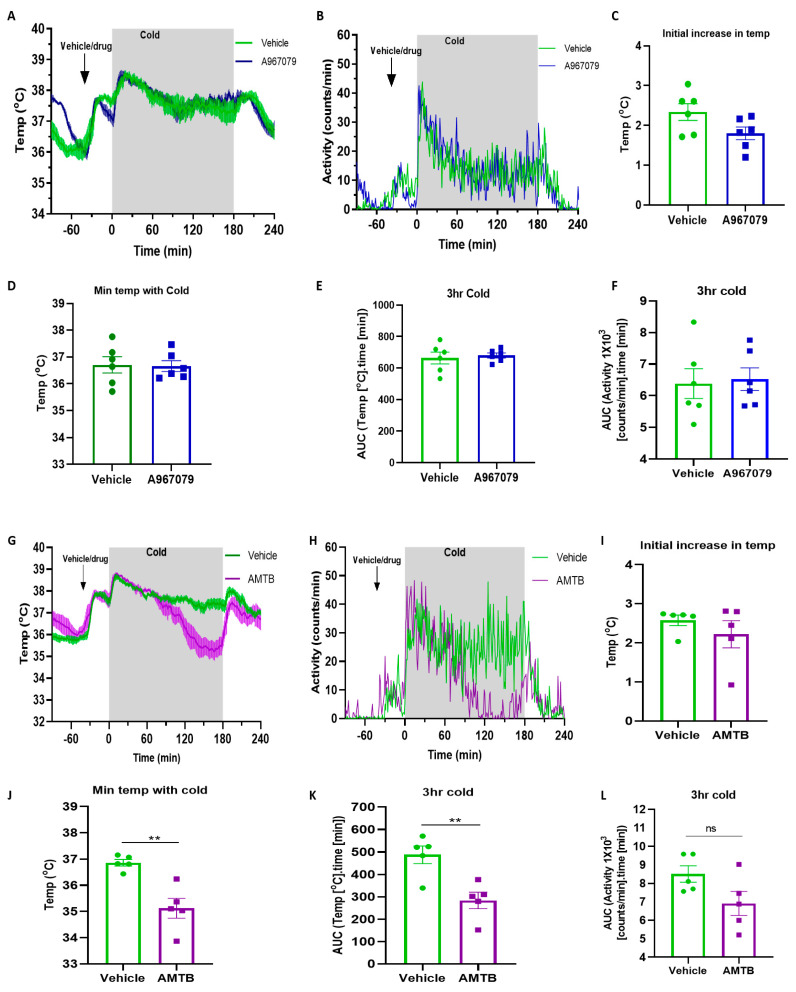
The effect of environmental cold in the presence of TRPA1 (**A**–**F**) and TRPM8 antagonist (**G**–**L**) in C57BL/6. The graphs represent (**A**) the mean core body temperature and (**B**) the mean activity changes during 3 h of cold (4 °C) exposure in a climatic chamber in the presence of TRPA1 antagonist A967079 (100 mg/kg) that was given orally 30 min prior to the cold treatment (*n* = 6). (**C**) The initial temperature increase at the start of cold exposure in the climatic chamber; (**D**) the minimum temperature achieved during 3 h of cold exposure; (**E**) the area under the curve (AUC) analysis of the temperature during the 3 h of cold treatment. (**F**) The area under the curve (AUC) analysis of the activity during 3 h of cold treatment. The graphs represent (**G**) the mean core body temperature and (**H**) the mean activity changes during 3 h cold (4 °C) exposure in a climatic chamber in the presence of TRPM8 antagonist AMTB (10 mg/kg) that was given orally 30 min prior to the cold exposure (*n* = 5). (**I**) The initial temperature increase at the start of cold exposure in the climatic chamber; (**J**) the minimum temperature that was achieved during 3 h of cold exposure; (**K**) the area under the curve (AUC) analysis of the core body temperature during 3 h of cold treatment; (**L**) The area under the curve (AUC) analysis of activity during 3 h cold treatment. All the data are presented as the mean and error bars indicate S.E.M. ** *p* < 0.01, ns = non-significant. Two-tailed Student’s *t*-test.

After confirming the effectiveness of oral treatment of the TRPA1 and TRPM8 antagonists, we investigated the pharmacological blockade of the two receptors on core body temperature during cold exposure. After 1 h of baseline recording, the mice were given Nutella that as mixed with an antagonist, which they consumed immediately. This caused some activity and increased temperature change, which was expected but it is substantially less than the disturbance that we have previously observed and investigated in depth before [32] following restraint (needed for drug administration when given by injection). The mice were moved into the cold chamber 30 min after the oral treatment for 3 h, followed by 1 h of recovery at room temperature. The movement into the climatic chamber caused a rapid increase in the temperature, as expected (Figure 4A,C), and increased the activity (Figure 4B). However, the TRPA1 antagonist had no effect on this compared to vehicle-treated mice. The mice maintained a high core body temperature throughout the 3 h of cold exposure but the TRPA1 antagonist had no effect on this response (Figure 4D,E).

We then investigated the administration of the TRPM8 antagonist. The mice showed a similar early increase in temperature and activity following the Nutella treatment, but this was similar in both the vehicle- and AMTB-treated mice. The move to the cold climatic chamber caused a rapid increase in the temperature and activity (Figure 4F,G) and the vehicle-treated mice maintained a higher core body temperature throughout the 3 h of cold exposure. In the AMTB-treated mice, however, although there was the initial increase in the temperature, their core body temperature slowly declined over the 3 h of cold exposure (Figure 4F) as shown by the area under the curve (Figure 4J), suggesting AMTB-treated mice are unable to maintain increased core body temperature in response to cold. The vehicle-treated mice achieved a minimum temperature of 36.8 ± 0.1 °C during cold exposure whereas the AMTB-treated mice achieved a minimum temperature of 35.0 ± 0.8 °C (*p* < 0.01 vs. vehicle-treated mice, Figure 4J). The activity graphs were analysed by the area under the curve to determine if a statistical difference was shown. However, the trend that was shown in mice in the AMTB treatment group did not reach significance (Figure 4H) and was not significantly different to that of mice given the vehicle (Figure 4L). Additionally, we examined the effect of AMTB on mice that were kept at room temperature in our environmental chamber. Under these circumstances, we saw no difference in responses (Supplementary Figure S2).

## 3. Discussion

TRPA1 and TRPM8 receptors have important roles in the molecular sensing of environmental cold but their precise mechanisms remain unclear. We have been interested in the effect of pharmacological antagonists on the maintenance of a healthy response to the cold, principally concentrating on the vascular response to local cold exposure in skin to date [9,34,35]. Here, we have extended our studies to whole body cooling. Our results show that after establishing a non-invasive and relatively stress-free mode of drug administration, there was little effect of the TRPA1 antagonist during cold exposure. This lack of effect was observed even though the antagonist that was used was able to inhibit TRPA1-mediated vasodilator activity and the sensitivity of TRPA1 to act as a primary cold sensor in the acute vascular cold response to local cooling in mice, as previously shown [9]. By comparison, the TRPM8 antagonist caused a significant decrease in the core body temperature as the cold-exposure progressed.

Firstly, we investigated the response of the core body temperature of naive mice, through use of telemetry temperature probes which allowed real time temperature recording from the peritoneal cavity and additionally monitors locomotion/movement activities. The results show that a cold environment, as made feasible by our environmental cold chamber, caused the mice to raise their body temperature over the duration of 1–3 h, presumably to combat the cold temperatures. This was associated with a trend towards increased activity, but this did not reach statistical significance.

Knowledge that humans, as well as mice, have a physiologically functional BAT [36,37,38] prompted us to investigate if the characteristic UCP-1 increase upon environmental cold exposure is associated with differences in TRPM8 protein expression in BAT. UCP-1 is the primary influencer of cold-mediated non-shivering thermogenesis and, thus, essential for maintaining the body temperature during cold exposure [4,39]. TRPM8 has been localised to BAT in some [21,40] but not all studies [41]. Of note, the TRPM8 agonists menthol- and icilin-induced thermogenesis was linked to weight loss [21,41]. Here the presence of TRPM8 in BAT was detected via RT-PCR and Western blotting. We, perhaps surprisingly, did not see an increase in the mRNA expression with cooling. However, with Western blotting for the TRPM8 protein using an established anti-TRPM8 antibody [21,30,42], we observed an increase in protein expression. Potentially, the difference could be due to the TRPM8 mRNA having been converted to protein by the time of our tissue collection. Ma et al. have shown that exogenous stimulation with chronic dietary TRPM8 agonist is linked directly to UCP-1 uncoupling, providing the link with potential weight loss, whilst these results are supported by more recent studies in adipocytes, where activation of TRPM8 was associated with adipocyte browning [43]. Our results, concentrating on cold exposure, support the concept that UCP-1 upregulation in BAT occurs with cold exposure which induces thermogenesis to produce heat and counter cold stimuli. The upregulation of TRPM8 in BAT with cold exposure suggests TRPM8 potentially has a role in thermogenesis. To support this theory, we carried out experiments with TRP antagonists. Before using the antagonists in the cold exposure study, we determined the selectivity of A967079 and AMTB for TRPA1 and TRPM8 channels, respectively. A967079 inhibited the TRPA1 agonist, cinnamaldehyde, induced an increased ear blood flow, and AMTB inhibited the TRPM8 agonist, menthol, and induced an increased ear blood flow. Furthermore, the selectivity of AMTB was investigated by examining the effect on ear blood flow that was induced by the TRPV1 agonist, capsaicin. AMTB had no inhibitory effect on capsaicin (Supplementary Figure S1).

TRPM8, which senses cool temperatures (e.g., <27 °C), is known to also reduce cold sensitivity and to be involved in the vascular cold response. Most studies to date have investigated the effect of TRPM8 in cold pain sensitivity in chemically-induced pain (e.g., oxaliplatin), where TRPM8 expression is increased [41]. As TRPM8 KO mice have been shown to exhibit decreased levels of UCP-1 [23], we chose to study pharmacological antagonism in this study where the baseline levels at the start of the experiment would be similar. Previous evidence has suggested that a TRPM8 antagonist acts peripherally to decrease the body temperature via peripheral mechanisms together with a decreased BAT thermogenic index [22]. We have shown that the TRPM8 antagonist can reduce the acute vascular cold response in the mouse paw in a model involving a cold probe [31]. In that study that was carried out in anaesthetised mice, drug administration via intra-peritoneal injection was performed. However, in whole body cold exposure in conscious mice, an improved administration protocol for drug administration is required as the procedure of moving and picking up mice is associated with core body blood pressure changes which, in turn, influences the core body temperature [42]. In this study, to enable administration of the TRPA1 and the TRPM8 antagonist without stress, the mice received a spread of a favoured food which they readily consumed. Using this regime, we determined that the addition of a TRPM8 antagonist (half-life 1.3 h) had a strong effect in influencing the body temperature during cold exposure. This suggests that the TRPM8 antagonist prevents the normal response to environmental cooling under situations where TRPA1 is unable to. We also investigated if the TRPM8 antagonist AMTB affected the body temperature response during room temperature (RT) exposure. The result showed that AMTB had no effect on the core body temperature and activity during room temperature exposure. This suggests that the TRPM8 signalling is cold-specific (Supplementary Figure S2). Our data are in keeping with the concept that TRPA1 has no effect and that TRPM8 influences the cold response. The data showed an inhibition of the rise in core body temperature during 3 h of cold exposure in the presence of the TRPM8 antagonist. In a murine model, a study showed an increased expression of TRPM8 and TRPA1 in the hypothalamus with 3 h of cold (4 °C) treatment [43]. Therefore, the upregulation of TRPM8 may be a protective positive feedback that kick-starts in cold environments to increase thermogenesis in BAT to produce heat. Current limitations of the present study are that we are not in a position to extend the studies through further molecular studies or use of conditional knockout mice.

## 4. Materials and Methods

### 4.1. Animals

Male CD1 and C57BL/6 mice (<3 months of age) were used which were purchased from Charles River (Kent, UK). All the mice were housed in a well-controlled environment consisting of rooms which were temperature-regulated with an ambient temperature of 22 °C, including a 12 h light and dark cycle and access to standard chow diet and water ad libitum. All the experiments were approved by the Animal Care and Ethics committee at King’s College London, in addition to the regulations that were set by the UK Home Office ‘Animals (Scientific Procedures)’ Act 1986. The experimental design was performed to allow the use of animals to fit with NC3Rs initiative by adhering to the ARRIVE guidelines.

### 4.2. Full-Field Laser Perfusion Imager (FLPI) of Cutaneous Blood Flow in the Ear

The cutaneous blood flow in the mouse ear was recorded as previously reported [44] with minimal adjustments. A967079 (100 mg/kg) or vehicle (10% DMSO, 10% Tween) with 200 µL Nutella spread was given to mice 30 min before the start of the blood flow recording. The mice readily ate this whether in the presence or absence of the vehicle ± antagonists and no adverse effects were observed. The mice were anaesthetised with isoflurane gas (5% with oxygen as the carrier gas) for induction, followed by 1.8% isoflurane for maintenance. To ensure that the mice maintained their core body temperature, they were placed on a heating mat which was set at 37 °C. A full-field laser perfusion imager (FLPI, Moor Instruments, Axminster, UK) was used to record the cutaneous blood flow in the ears. An initial 5 min baseline blood flow was recorded. The recording was then paused to allow the topical application of TRPA1 agonist 10% cinnamaldehyde (CA) or TRPM8 agonist 10% menthol or TRPV1 agonist 10% capsaicin to both sides of the ipsilateral right ear, with the contralateral ear receiving the vehicle treatment 10% DMSO. The recording was resumed for 30 min to observe the changes in blood flow in response to CA and menthol.

### 4.3. Radiotelemetry and Whole Body Cold Exposure

The radiotelemetry device, TAT11-PAC-10 (Data Sciences International, DSI, Minnesota, USA), was used to measure the core body temperature and activity of the mice. For activity, the probe measures any movement in counts per minute that causes a disturbance to the telemetry probe signal. This is computed by the telemetry matrix and software (Data Sciences International). The surgical procedure was carried out in anaesthetized mice using aseptic conditions with 1.8% isoflurane (carried in oxygen) and a homeothermic monitoring system to maintain the core body temperature at 37 °C. 50 µg/kg meloxicam (Metacam, Boehringer Ingelheim, Duluth, Georgia) was given i.p. as an analgesic. Roughly 1 cm^2^ area of abdominal region was shaved and disinfected with iodine solution. A small incision (~1 cm) was made in the midline of the peritoneal region of the shaved abdominal cavity and the telemeter device was implanted. After insertion of the probe, the wound was irrigated with sterile saline and both the abdomen muscle and skin closed with a surgical braided silk (5.0, waxed, Pearsalls sutures, Pennsylvania, USA). The animals were kept in a warm box (28 °C) for 24 h for initial recovery and to monitor signs of pain and infection after which they were left to recover for another 6 days in a normal housing environment in their home cage.

To record the core body temperature and activity of the telemetered mice, 1.5 h of baseline was first recorded in their home cage with food and water. After the baseline recording was over, the mice were moved into a new cage without food. For cold exposure, the mice in their new cage were moved into a climatic chamber that was set at 4 °C or at room temperature (RT) for the control mice. Following the cold or RT treatment, the mice were moved back into their home cage and their recovery was measured at room temperature for another 1 h. The data were recorded in a continuous fashion using DSI Dataquest software and analysed in Microsoft Excel and Graphpad Prism 8.

### 4.4. Western Blotting

Brown adipose tissue (BAT) was collected from C57BL/6 mice immediately following either the 3 h room temperature or cold exposure (4 °C) and snap-frozen in liquid nitrogen and stored at −80 °C. The tissue was lysed with SDS lysis buffer which was made up with inhibitors for both phosphatases and proteases (1 tablet per 10 mL, #4693159001 + #4906845001, Sigma-Aldrich, Gillingham, UK). The tissue was then homogenised using a tissue lyser (#85300, Qiagen, Manchester, UK). The protein concentration of the BAT tissue was determined using the Bradford protein dye-binding assay (#5000113 + #5000114, Bio-Rad, Hertfordshire, UK). When probing for UCP-1, 10 µg of protein was loaded, whereas 50 µg was loaded when probing for TRPM8. The protein separation was performed by electrophoresis in an SDS-polyacrylamide gel which was then transferred using the semi-dry method, onto PVDF membranes. The membrane was incubated in a blocking buffer that was made up of 5% BSA in phosphate-buffered saline-tween (PBS-T) (0.1% Tween, Sigma-Aldrich, Gillingham, UK). The UCP-1 blot was blocked for 1 h in RT whereas the TRPM8 blocked for 2.5 h as per the manufacturer’s instruction. The membranes with the primary antibodies against UCP-1 (1:5000 dilution, #AB10983, Abcam, Cambridge, UK) and TRPM8 (1:200 dilution, #ACC-049, Alomone, Jerusalem, Israel) were incubated for 1 h at RT and overnight at 4 °C, respectively. Following the washing step with PBS-T, the membranes were probed with a secondary antibody (Horseradish peroxidase conjugated) (1:2000 dilution, #AP132P, Sigma-Aldrich, Gillingham, UK) for 1 h at RT. The enhanced chemiluminescence (ECL, Piercenet, Thermo Fisher Scientific, Waltham, MA, USA) was used for the visual development of the membranes inside a gel doc system. The bands were normalised to housekeeping genes of α-tubulin (1:2000, #MAB1864, Merck Millipore, Hertfordshire, UK) and β-actin (1:2000, #A5441, Sigma-Aldrich, Gillingham, UK). Quantitative Western blot analysis was performed using Image J (NIH, Washington, DC, USA).

### 4.5. Polymerase Chain Reaction

Real-time PCR (RT-PCR) was used to quantify the mRNA change in the brown adipose tissue from the room temperature (RT) and cold treated C57BL/6 mice. After the 3 h of RT or cold treatment, the mice were culled and the BAT was collected and snap-frozen in liquid nitrogen and stored at −80 °C. The total RNA was isolated and purified using the RNeasy Mini Kit (#74104, Qiagen, Manchester, UK). The RNA was reverse transcribed to cDNA using the SuperScript ViLO cDNA synthesis kit (, #11754050, Thermo fisher scientific, Manchester, UK). qPCR was performed with 10 ng of cDNA using the PowerUp SYBR Green master mix kit (#A25780, Thermo fisher scientific, Manchester, UK) in 7900HT Real-Time PCR machine (Applied Biosystems, Waltham, MA, USA). The melting curve analysis was performed after each PCR run to check primer specificity and qPCR analysis was performed using delta-delta CT method where the change was shown as fold change that was normalized to the average of three housekeeping genes. The primers that were used for UCP-1 were (Fwd: CCGAAACTGTACAGCGGTCT, Rev: CCGAGAGAGGCAGGTGTTTC) and TRPM8 (Fwd: TTGTATTCCGGCTCCACTCTTC, Rev: AGTTCCTGCTGACGGTGAAAA).

### 4.6. Drugs and Reagents

Cinnamaldehyde (CA) (#W228613, Sigma-Aldrich, Gillingham, UK, >95% purity), capsaicin (#M2028, Sigma-Aldrich, Gillingham, UK >95%), and menthol (#2772, Sigma-Aldrich, Gillingham, UK >99%) was prepared as a 20 μL solution with a final topical dose of 10%. Cinnamaldehyde, capsaicin, and menthol were dissolved in an ethanol vehicle solution which contained 10% DMSO as a solvent. The vehicle solutions that were used were 10% DMSO in ethanol. A967079 (#A-225, Alomone, Jerusalem, Israel >99% purity) powder was dissolved in 10% DMSO + 10% Tween-80 in saline and given at a final dose of 100 mg/kg. The vehicle for A967079 was 10% DMSO + 10% Tween-80 that was dissolved in saline. N-(3-Aminopropyl)-2-[(3-methylphenyl)methoxy]-*N*-(2-thienylmethyl)benzamidehydrochloride (AMTB) (#A-305, Alomone, Jerusalem, Israel >97% purity) was dissolved with 10% DMSO in saline to produce a final dose of 10 mg/kg. The A967079, AMTB, and vehicle solution were then given orally via a mixture with 200 µL chocolate-spread Nutella (containing 13% hazelnuts) Ferrero SpA, Alba, Italy), which the mice readily consumed.

### 4.7. Statistical Analysis

All the data that are presented are the mean ± S.E.M (standard error of mean) unless otherwise stated. The data were tested for normal distribution using Shapiro-Wilk normality test. Statistical analysis was performed using an unpaired two-tailed Student’s *t*-test for two data groups, two-way ANOVA, followed by Tukey post hoc test for multiple groups. *p* < 0.05 was considered statistically significant. MS-Excel and GraphPad Prism Ver 8.0 was used for statistical analysis.

## 5. Conclusions

We conclude that our results show the lack of influence of TRPA1 in environmental cold sensing to induce thermogenesis under situations where its role as a peripheral vascular cold sensor is clear; to mediate cutaneous vasoconstriction. This leads us to question the physiological purpose of the vascular cold response and its relative importance in protecting against the environmental temperature. One possibility is that the major role of the vascular cold response is to limit peripheral heat loss at the cutaneous level. Here, its role is linked to more pathological situations where continued vasoconstriction will lead to damage to the skin, as observed in cold-induced injuries that are associated with chilblains, Raynaud’s disease, and potentially other conditions where the peripheral vasculature is compromised, such as diabetes.

## Data Availability

Data is contained within the article and Supplementary Material.

## Supplementary Materials

The following supporting information can be downloaded at: https://www.mdpi.com/article/10.3390/ph15010042/s1, Figure S1: Determining the effect of AMTB given orally in Nutella spread on TRPV1 signaling in CD1 mice, Figure S2: The effect of TRPM8 antagonist AMTB (10mg/kg) given orally in Nutella spread 30 min prior to start of room temperature (RT) exposure (A-F).
