# Supplementary material for: Influence of Cold-TRP Receptors on Cold-Influenced Behaviour
Source: Pharmaceuticals (Basel). 2021 Dec 28;15(1):42. doi: 10.3390/ph15010042 (PMC8781072; doi:10.3390/ph15010042)
Supplement: Supplementary file 1 [file pharmaceuticals-15-00042-s001.zip › pharmaceuticals-1420050-supplementary.pdf]

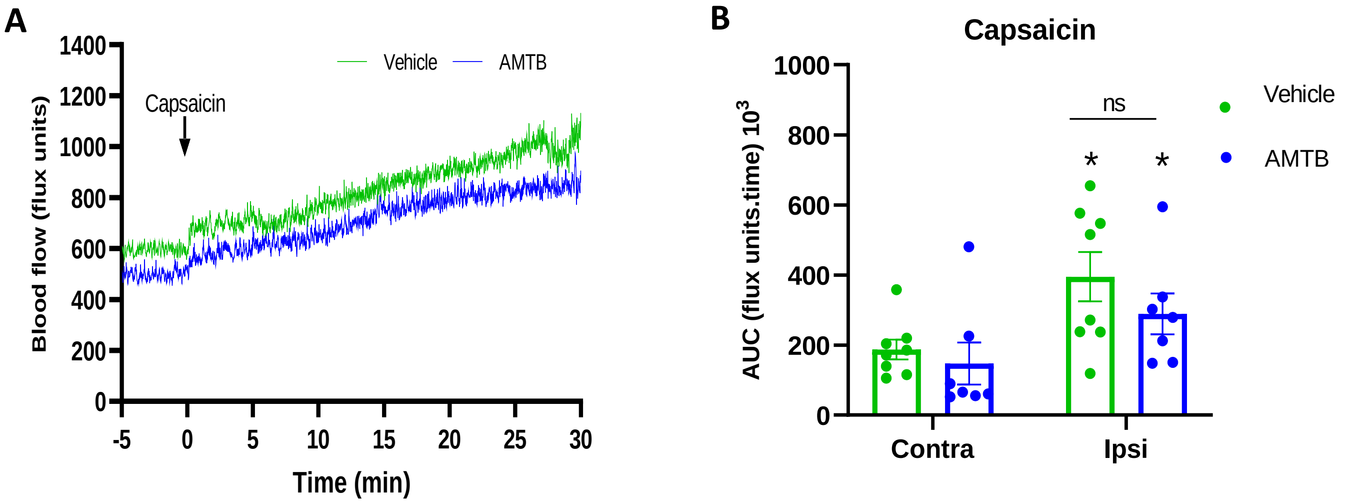

Supplementary Figure S1. Determining the effect of AMTB given orally in Nutella spread on TRPV1 signalling in CD1 mice. (A) Graph shows the mean blood flow response to topical application of the TRPV1 agonist capsaicin (10%) on the ipsilateral ear in the presence of the TRPM8 antagonist, AMTB (10mg/kg), given orally 30min prior to capsaicin application. (B) Area under the curve (AUC) analysis of blood flow increase after vehicle (10% DMSO) or capsaicin treatment in the presence of AMTB (n=7-8). *$p<0.05$ against contralateral ear, ns= non significant. (Two-way ANOVA, Tukey's post hoc test)

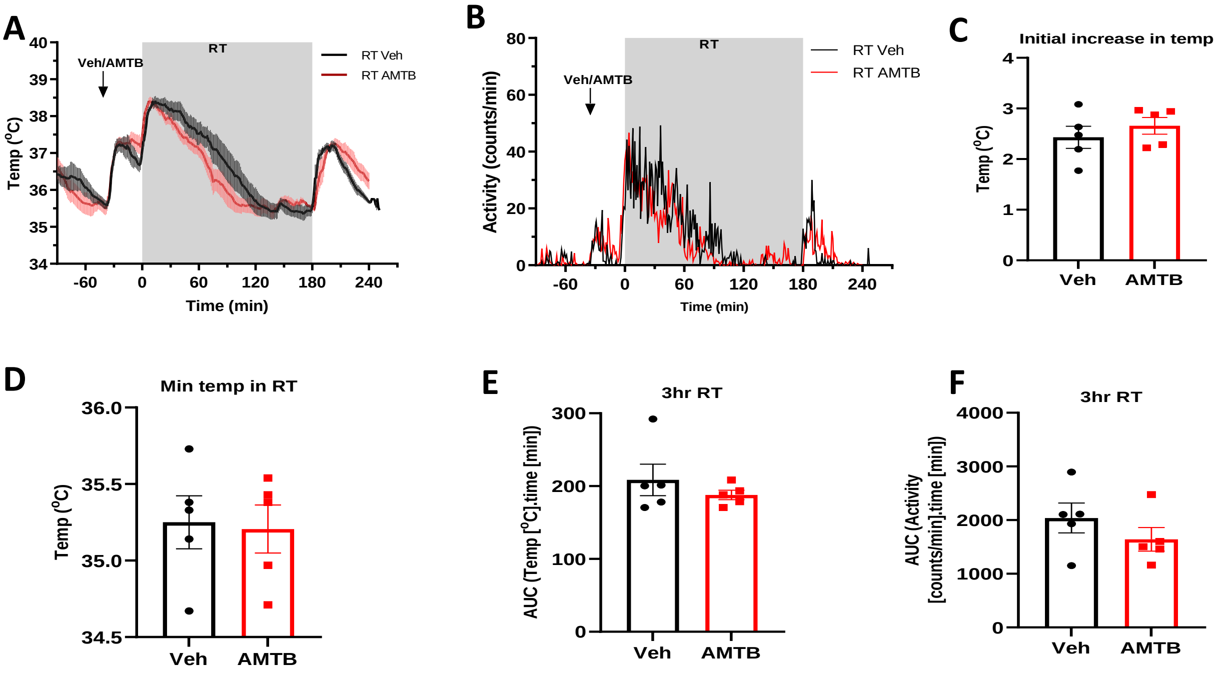

Supplementary Figure S2. The effect of TRPM8 antagonist AMTB (10mg/kg) given orally in Nutella spread 30 min prior to start of room temperature (RT) exposure (A-F). Graph shows (A) the mean core body temperature and (B) the mean activity changes in C57 mice during 3hr of room temperature (RT) (n=5). (C) the initial temperature increase at the start of RT exposure (D) minimum temperature achieved during 3hr of RT exposure (E) area under the curve (AUC) analysis of temperature during 3hr of RT treatment. (F) area under the curve (AUC) analysis of activity during 3hr of RT treatment. Results are for n= 5 mice. All data are presented as mean and error bars indicate s.e.m. Two-tailed Student's t-test.
